# Exercise Training Results in Lower Amyloid Plaque Load and Greater Cognitive Function in an Intensity Dependent Manner in the Tg2576 Mouse Model of Alzheimer’s Disease

**DOI:** 10.3390/brainsci10020088

**Published:** 2020-02-08

**Authors:** Riya Thomas, Scott D. Zimmerman, Kayla M. Yuede, John R. Cirrito, Leon M. Tai, Benjamin F. Timson, Carla M. Yuede

**Affiliations:** 1 Department of Biomedical Sciences, Missouri State University, Springfield, MO 65897, USA; riyarachtom@hotmail.com (R.T.); scottzimmerman@missouristate.edu (S.D.Z.); bentimson@missouristate.edu (B.F.T.); 2 Department of Neurology, Washington University School of Medicine, St. Louis, MO 63110, USA; kayla.yuede@slu.edu (K.M.Y.); cirritoj@wustl.edu (J.R.C.); 3 Hope Center, Washington University School of Medicine, St. Louis, MO 63110, USA; 4 Alzheimer’s Disease Research Center, Washington University School of Medicine, St. Louis, MO 63110, USA; 5 Department of Anatomy and Cell Biology, University of Illinois at Chicago, Chicago, IL 60607, USA; leontai@uic.edu

**Keywords:** Alzheimer’s disease, exercise training, amyloid plaque, cognitive function, Tg2576 mice

## Abstract

Three months of exercise training (ET) decreases soluble Aβ_40_ and Aβ_42_ levels in an intensity dependent manner early in life in Tg2576 mice (Moore et al., 2016). Here, we examined the effects of 12 months of low- and high- intensity exercise training on cognitive function and amyloid plaque load in the cortex and hippocampus of 15-month-old Tg2576 mice. Low- (LOW) and high- (HI) intensity ET animals ran at speeds of 15 m/min on a level treadmill and 32 m/min at a 10% grade, respectively, for 60 min/day, five days/week, from 3 to 15 months of age. Sedentary mice (SED) were placed on a level, non-moving, treadmill for the same duration. ET mice demonstrated a significantly lower amyloid plaque load in the cortex and hippocampus that was intensity dependent. Improvement in cognitive function, assessed by Morris Water Maze and Novel Object Recognition tests, was greater in the HI group compared to the LOW and SED groups. LOW mice performed better in the initial latency to the platform location during the probe trial of the Morris Water Maze (MWM) test than SED, but not in any other aspect of MWM or the Novel Object Recognition test. The results of this study indicate that exercise training decreases amyloid plaque load in an intensity dependent manner and that high-intensity exercise training improves cognitive function relative to SED mice, but the intensity of the LOW group was below the threshold to demonstrate robust improvement in cognitive function in Tg2576 mice.

## 1. Introduction

Alzheimer’s disease (AD) is a chronic neurodegenerative disease with growing worldwide impact. Environmental and pharmaceutical interventions have been investigated as strategies attempting to delay or slow the progression of AD. One such environmental intervention is physical activity. Physical activity is broadly defined as any movement of the body resulting from muscle contraction that elevates total body energy expenditure above that of rest [1] and has long been associated with a healthy lifestyle. PA covers a wide range of intensities, frequencies, durations, and types and can be planned or unplanned and chronic or acute [2]. All of these contribute to the degree to which it contributes to improved health. Typically, there is a threshold level of PA below which health is not improved and above which health is improved in a dose-dependent manner [2].

Chronic physical activity has been shown to reduce cognitive decline and AD risk in humans [3,4,5,6,7,8]. Treadmill running has been used for the past decade to investigate the effects of chronic physical activity on amyloid plaque deposition, a classic hallmark of AD, and cognitive function in AD transgenic mouse models. The results of these studies have been equivocal, however, with many [9,10,11,12,13,14,15], but not all [16,17] reporting amyloid plaque load decreased in mice subjected to treadmill running. Behavioral analyses conducted with AD transgenic mice following treadmill running regimens have also yielded inconsistent results with many [9,10,11,12,17,18,19,20] reporting improved performance on a variety of behavioral tests, whereas others [14,15,16] found no change in behavioral performance following a treadmill running regimen.

One possible factor for these disparate findings is a wide range in speed and distance run by the mice in different studies. Exercise training quantity, expressed by intensity, duration, and frequency, has been demonstrated to positively impact cardiovascular disease risk in a dose-dependent manner [21]. It is likely that chronic neurological disorders, such as AD, whose risk is reduced by physical activity respond in a similar dose-dependent manner as cardiovascular disease. To date, no study has investigated the dose-dependent effects of chronic physical activity on amyloid plaque deposition and cognitive function in a transgenic mouse model of AD.

Soluble Aβ exists in the brain interstitial fluid (ISF) throughout life as a result of sequential cleavage of the amyloid precursor protein by β- and γ-secretase and aggregates into soluble oligomers and insoluble plaque in a concentration dependent manner [22,23,24]. It follows, that implementing strategies early in life and maintaining them throughout life, to keep soluble Aβ levels low in the brain would slow its aggregation possibly delaying the onset and slowing the progression of AD. Recently, Moore et al. [25] reported that soluble Aβ_40_ and Aβ_42_ were decreased in the cortex and hippocampus in an exercise training dose-dependent manner in Tg2576 mice following a three month treadmill running program ending at six months of age, well before the onset of plaque deposition in this model. The purpose of this study was to extend the study of Moore et al. [25] and determine whether or not continuing the exercise training regimen for 12 months in Tg2576 mice (from 3–15 months of age) would result in a dose-dependent decrease in amyloid plaque levels and improvement in cognitive function.

Our results demonstrate that exercise training reduces amyloid plaque load in an intensity dependent manner in male Tg2576 mice. Further, cognitive function was increased in high-intensity exercise-trained male mice in this study relative to low-intensity exercise-trained and sedentary mice. However, cognitive function was similar in mice subjected to low-intensity exercise training compared with sedentary controls indicating there is a physical activity threshold below which improvement in cognitive function is not realized. We used only male mice in this study to avoid a sex effect, it is possible female mice would not respond in a similar manner.

## 2. Materials and Methods

### 2.1. Animals and Experimental Design

**Figure 1:**
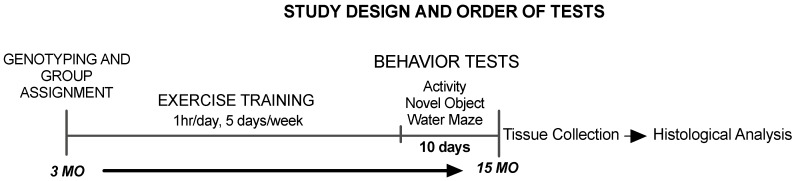
Experimental Design. Age of exercise training and order of behavior tests.

A total of 26 male Tg2576 mice from our breeding colony were used for these experiments which were approved by the Missouri State University Animal Care and Use Committee. Animals were housed individually in Optimize ventilated cages and provided food and water ad libitum. Mice were housed individually to control for the tendency of male mice to fight when housed with other males. Fighting would lead to a stressful environment which could adversely affect study results. Mice were randomly assigned to one of three groups similar to those of our previous study [25]. A low-intensity running group (LOW) ran on a level treadmill at a speed of 15 m/min and a high intensity running group (HI) ran at a speed of 32 m/min on a 10% grade. Treatment was performed for 60 min/day, five days/week, for 12 months beginning at three months of age and lasting until 15 months of age. Motivation to run was provided by a small electrical shock grid at the back of the treadmill. Mice were carefully observed during all running sessions and were compliant with the running protocol throughout the study. Sedentary (SED) mice were removed from their cages and placed on a stationary treadmill for the same duration and frequency as the mice undergoing treatment. Two weeks before the end of the exercise training period, behavioral function was assessed (Figure 1). All exercise training and behavior testing was conducted during the dark phase of the light/dark cycle. Due to a scheduling error, a group of mice (5 SED, 1 LOW and 2 HI) were not tested in open field and started directly with the Novel Object Recognition protocol, and water maze data were not collected for 5 mice (4 SED, 1 HI) that refused to swim during the cued trials sessions. Body weights were measured at the end of the study and were not significantly different between groups (SED = 32.75 ± 1.71g; LOW = 30.44 ± 2.19g; HI = 31.6 ± 1.82g) One-way ANOVA (F(2,15) = 1.924, *p* = 0.1803).

### 2.2. Tissue Preparation

Animals were sacrificed by isoflurane at the end of the 12 month treatment regimen. Brains were promptly removed and immersed in 4% paraformaldehyde for 24 h then transferred to a 30% sucrose solution and stored for later histological analysis. Brain sectioning and amyloid imaging was not performed on 3 SED, 4 LOW, and 2 HI mice due to tissue damage during the processing procedure. The soleus muscle was removed from each hindlimb, snap frozen on dry ice, and stored at −80 °C for citrate synthase analysis.

### 2.3. Soleus Muscle Citrate Synthase Analysis

Citrate synthase (CS) activity was assayed from a cohort of mice which included SED (*n* = 4), LOW (*n* = 9), and HI (*n* = 5). Following sonication in CellLytic MT buffer, soleus muscle CS activity was assessed using a Sigma Aldrich Citrate Synthase Enzyme Activity kit. Absorbance was measured at 412 nm on a BioTek (Winooski, VT, USA) Epoch plate reader and values for left and right soleus muscles of each animal were averaged. Total protein was quantified using the Thermo Scientific^TM^ Pierce^TM^ BCA^TM^ protein assay.

### 2.4. Brain Tissue Sectioning and Amyloid Plaque Staining

Brain hemispheres were mounted in Tissue-Tek OCT compound from SED (*n* = 6, LOW (*n* = 6), and HI (*n* = 5) mice. Then, 40 µm thick coronal sections were cut using a cryostat (Microm HM550, ThermoFischer Scientific, Walldorf, Germany). Sections were fixed and stained using mHJ3.4B, a biotinylated reporter antibody specific to N-terminus of Aβ. After incubation with streptavidin-HRP, Aβ plaques were detected by DAB and viewed using light microscopy. Plaque area in the cortex and hippocampus was determined using NIH Image J software. Images were captured for plaque staining at 40× magnification. Plaque was also assessed by Thioflavin S staining. Slides were incubated in filtered 1% aqueous Thioflavin-S (Sigma, St. Louis, MO, USA) for 5 min, dehydrated twice in 70% ethanol for 5 min, and washed twice in PBS for 2 min. Mosaic images were captured for amyloid plaque staining at 10× magnification.

### 2.5. Open Field Test

Two weeks before the end of the treatment period SED (*n* = 4), LOW (*n* = 9), and HI (*n* = 5) mice were placed in the center of a white box (138.5 × 30 cm surface, 30 cm high) and allowed to move freely for 10 min. Distance travelled and distance in the center of the arena were measured using Any-Maze software.

### 2.6. Novel Object Recognition Test

The day following the Open Field Test SED (*n* = 9), LOW (*n* = 10), and HI (*n* = 7) mice were acclimated to the test area for 20 min. The next day each mouse was subjected to a sample trial and a test trial. The objects consisted of 6 each of glass votive size candle holders and similar sized small metal baskets that had been determined to be of equal interest in a broad object screening with a test group of mice in pilot studies [15]. Presentation of the votive or basket as the novel object and side of novel object was counterbalanced across groups. During the sample trial, the mouse was exposed to two identical objects placed in opposite corners of the test area for 10 min. The mouse was then returned to his cage for 50 min. Following the delay, the mouse was put back into the test area for 10 min with one of the objects from the sample trial replaced with a novel object. The test area and objects were cleaned with 70% ethanol after each trial. All sessions were recorded using Any-Maze software. Time spent investigating each object was timed manually from the recordings using a stopwatch. Active investigation was defined as the mouse facing the object within close proximity (2 cm) and/or touching the object with forepaws.

### 2.7. Morris Water Maze Test

MWM testing began the day following the completion of NOR and was conducted with SED (*n* = 5), LOW (*n* = 10), and HI (*n* = 6) mice in a 150 cm diameter pool divided into four equal quadrants. Two days of cued trials took place before beginning spatial learning trials to familiarize animals with the task and to determine differences in motivation or visual disturbances (data not shown). During cued trials, the platform was marked with a tennis ball attached to a steel rod. Four trials were administered per day with the platform cycled through each quadrant. Mice were started in the quadrant opposite the goal quadrant in the cued trials. Five days of place trials followed the cued trials with the “goal quadrant” containing a submerged platform and extramaze cues were used to facilitate spatial learning. Black shaped cues were placed on the walls surrounding the pool and mice were started in a different quadrant using a random start pattern. In both the cued and place trials latency was recorded with a maximum of 60 s allowed per trial. If the mouse did not find the platform within 60 s, it was led to the platform. In all cases mice were allowed to remain on the platform for 30 s. A single 60 s probe trial was conducted one hour following the final place trial where the platform was removed and latency to the former platform location and the time spent the goal quadrant were recorded. All Morris Water Maze trials were evaluated using Any-Maze video tracking system (Stoelting Co., Wood Dale, IL, USA).

### 2.8. Statistical Analysis

One-way ANOVA was used to determine whether or not there were significant effects of treatment on soleus muscle citrate synthase activity and plaque load in the cortex and hippocampus. Tukey’s post-hoc comparison was used to evaluate between group differences. Repeated-measures ANOVA was used to determine whether there were significant effects of treatment between objects in object recognition test with exercise treatment (SED, LOW, HI) as the between-subjects factor and the two objects (right, left; familiar, novel) as the within-subjects factor. Repeated-measures ANOVA was also used to determine whether there were significant effects of treatment status between place trial days in MWM with exercise treatment as the between-subjects factor and the five days of place trials as the within-subjects factor. One-way ANOVAs were performed to evaluate treatment effects on total investigation times in sample and test trials for object recognition and probe trials using MWM. One-way ANOVA was also used to analyze treatment effects on anxiety levels by total distance in the arena and time spent in the center zone in the open-field test. All analyses were performed using GraphPad Prism version 6 with statistical significance set at *p* < 0.05. If ANOVA suggested significant effects of group, Bonferroni *post-hoc* comparison was used to evaluate between group differences. All data are presented as the mean ± SEM.

## 3. Results

### 3.1. Increased Skeletal Muscle Citrate Synthase Activity (CS) is a Marker of Exercise Training Effects

**Figure 2:**
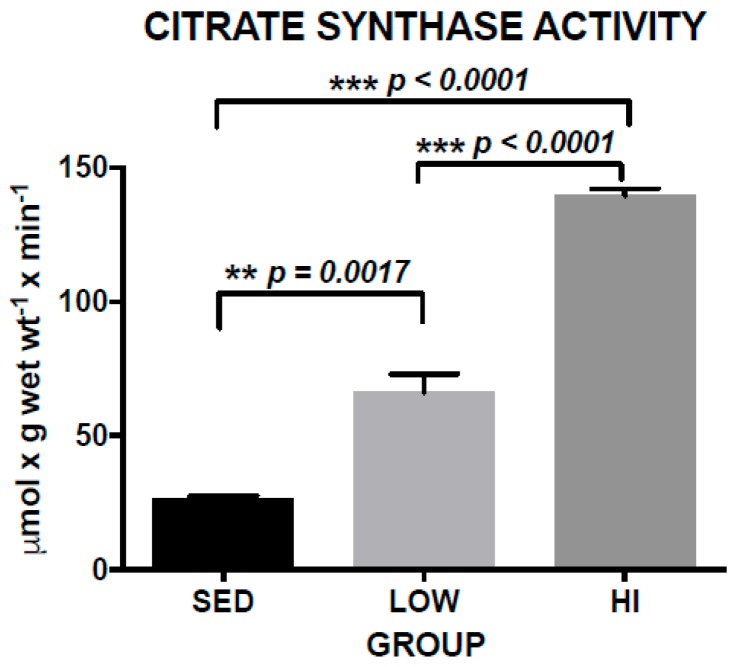
Soleus muscle citrate synthase activity in increased in a dose-dependent manner for sedentary (SED), low-intensity exercise-trained (LOW), and high-intensity exercise-trained (HI) Tg2576 mice. Values represent the mean ± s.e.m.

Exercise training is a specific form of chronic physical activity with the goal of improving physical fitness. Typically, exercise training consists of a planned regimen that includes a specified intensity, duration, frequency and mode of physical activity. The effect of an exercise training program can be assessed by quantifying some physiological parameter impacted. One such method of assessing the effect of a cardiovascular exercise training regimen, such as treadmill running, is to evaluate skeletal muscle oxidative capacity by quantifying a Krebs cycle or electron transport enzyme activity. CS is a Krebs cycle enzyme that we used to evaluate exercise training effects. The 12 month treadmill running program employed in this study had a large impact on CS activity. Mean CS activity in the LOW group was 149% greater than the SED group and the HI group CS activity was 110% greater than the LOW group (Figure 2). One-way ANOVA of CS activity shows a significant difference between groups (F(2,15) = 65.72, *p* < 0.0001). Post-hoc comparisons indicate that the HI group had significantly greater CS activity compared to LOW (*p* < 0.0001) and SED (*p* < 0.0001) groups, and that the LOW group had greater CS activity compared to the SED group (*p* = 0.0017). These data demonstrate a dose-dependent exercise training effect occurred in this study.

### 3.2. Exercise Training Reduces Amyloid Plaque Deposition in an Intensity-Dependent Manner in Tg2576 Mice

**Figure 3:**
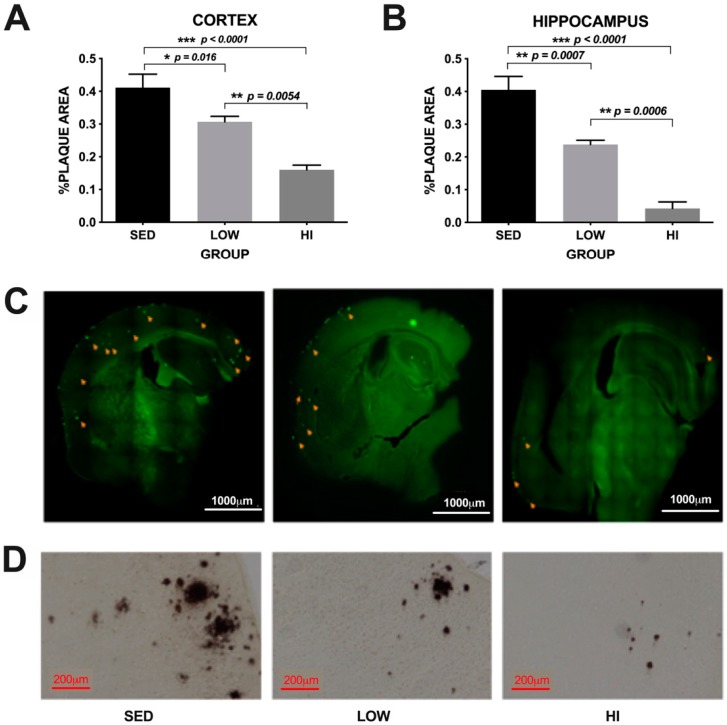
Amyloid plaque % area is decreased in an exercise training dose-dependent manner in both the cortex (Panel **A**) and hippocampus (Panel **B**) of Tg2576 mice following 12 months of treadmill running. Values represent the mean ± s.e.m. Panel **C** shows a representative image of amyloid plaque detected by Thioflavin S staining at 10× magnification. Panel **D** shows a representative image of amyloid plaque detected by DAB staining at 40× magnification.

Our earlier study [25] demonstrated that soluble Aβ was reduced by exercise training in an intensity-dependent manner when conducted from three to six months of age (prior to the time of amyloid plaque development) in Tg2576 mice. One goal of this study was to determine whether or not the same exercise training regimen conducted for 12 months, from three to 15 months of age (well beyond the time of amyloid plaque development in these mice), would reduce plaque in a similar intensity-dependent manner. Amyloid plaque area in the cortex was 46.8% and 60.2% lower in the HI mice compared to LOW and SED, respectively and 25.3% lower in LOW mice compared to SED (Figure 3A,C,D). In the hippocampus, amyloid plaque area was 79.2% and 87.6% lower in the HI mice compared to LOW and SED, respectively and 40.7% lower in LOW mice compared to SED (Figure 3B). One-way ANOVA of plaque load revealed significant differences between groups in the cortex (F(2,14) = 19.47, *p* < 0.0001) and hippocampus (F(2,14) = 39.76, *p* < 0.0001), with post-hoc comparisons showing mice in the HI group have significantly lower plaque load compared to mice in the LOW group in the cortex (*p* = 0.0054) and hippocampus (*p* = 0.0006), and compared to SED mice in both the cortex and hippocampus (*p* < 0.0001). Mice in the LOW group showed significantly less plaque load compared to SED mice in both the cortex (*p* = 0.016) and the hippocampus (*p* = 0.0007). These data demonstrate that exercise training intensity plays a significant role in reducing amyloid deposition in the Tg2576 mouse model of Alzheimer’s disease.

### 3.3. Exercise Training Does not Affect Locomotor Activity in Open Field Test

Differences in general locomotor activity and activity in the center of the open field can be an indicator of anxiety which could affect performance in other behavioral tests and thus is an important control in interpreting behavioral data. Total distance covered in the Open Field Test (One-way ANOVA F(2,15) = 0.4449, *p* = 0.6491) as well as distance in the center of the arena (One-way ANOVA F(2,15) = 0.0904, *p* = 0.914) were not different among groups and indicating that general activity levels or anxiety were not affected by the exercise training regimens employed in this study (data not shown).

### 3.4. High-Intensity Exercise Training Had Robust Effects on Spatial Learning and Memory in Tg2576 Mice, Whereas Effects Associated with Low-Intensity Exercise Training Were Subtle

**Figure 4:**
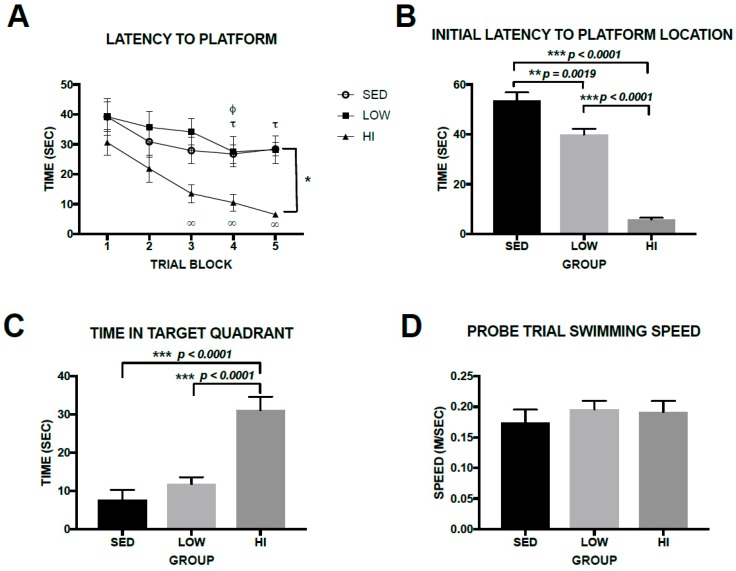
Exercise training intensity plays a role in Morris Water Maze performance in Tg2576 mice. Panel **A** shows latency to the platform during the place trials. Relative to day 1, latency was significantly faster in HI mice on day 3 (∞ *p* = 0.0003), day 4 (∞ *p* < 0.0001), and day 5 (∞ *p* < 0.0001). Relative to day 1, the LOW mice were significantly faster to the platform on day 4 (τ *p* = 0.0005) and day 5 (τ *p* = 0.0012) and the SED group was significantly faster to the platform on day 4 (φ *p* = 0.04). The HI group was significantly faster to the platform than the LOW group on day 5 (* *p* = 0.05). Panel **B** shows the initial latency to the platform location during the probe trial. Panel **C** depicts time in target quadrant during the probe trial. Panel **D** shows that swimming speed during the probe trial. Values represent the mean ± s.e.m.

Morris Water Maze is a common method of assessing spatial memory in rodents. Two-way Repeated-measures ANOVA of latency to locate platform data revealed significant main effects of GROUP (F(4,80) = 3.517, *p* = 0.0491) and TRIAL (F(4,80) = 18.02, *p* < 0.0001) as all three groups learned the platform location during the place trials (Figure 4A). The HI group learned the location of the platform faster than the LOW and SED groups. Analysis of simple main effects show that performance of the HI group improved significantly from day 1 to day 3, 4, and 5 (*p* = 0.0003, and *p* < 0.0001 for both 4 and 5, respectively). The LOW group significantly improved from day 1 to day 4 and 5 (*p* = 0.0005 and *p* = 0.0011, respectively) and the SED group improved from day 1 to day 4 (*p* = 0.0336). However, comparison between day 1 and day 5 in the SED group was not significant (*p* = 0.0893), suggesting minimal improvement from the beginning of spatial learning. Average swimming speeds during the learning trials were 0.1946 ± 0.064 m/s; 0.2137 ± 0.0523 m/s, and 0.1792 ± 0.038 m/s for the SED, LOW and HI groups, respectively. One-way ANOVA of average swimming speeds during the learning trials indicates no significant difference between groups (F(2,19) = 0.861, *p* = 0.4396). In the probe trial the HI group reached the platform location much faster (One-way ANOVA F(2,19) = 87.8, *p* < 0.0001) than the LOW (*p* < 0.0001) and SED (*p* < 0.0001) groups (Figure 4B) and spent much more time in the target quadrant (One-way ANOVA F(2,19) = 21.13, *p* < 0.0001) than the LOW and SED groups (both post-hoc comparisons *p* < 0.0001) (Figure 4C). The LOW group reached the platform location significantly faster than the SED group, but the time spent in the target quadrant did not differ significantly between the LOW and SED groups (*p* = 0.4849). Swimming speed during the probe trial was not different among groups (Figure 4D) indicating this was not a factor in latency differences among groups (One-way ANOVA (F(2,19) = 0.4344, *p* = 0.6539). Taken collectively, high-intensity exercise training had a clear and significant impact on spatial learning as assessed by Morris Water Maze. Mice in the HI group located the platform significantly faster on day 5 than LOW mice. They also had significantly shorter latency to the platform location and spent significantly more time in the target quadrant than LOW and SED mice during the probe trial. The effect of low-intensity exercise training on spatial memory is not robust as the LOW group mice had a significantly faster latency to the platform location in the probe trial than SED mice, but did not differ from SED mice in platform latency in the place trials nor did they differ from SED mice in time in the target quadrant in the probe trial.

### 3.5. High-Intensity Exercise Training Positively Impacts Recognition Memory in Tg2576 Mice as Assessed by a Novel Object Recognition Test, whereas, Low-Intensity Exercise Training Does not

**Figure 5:**
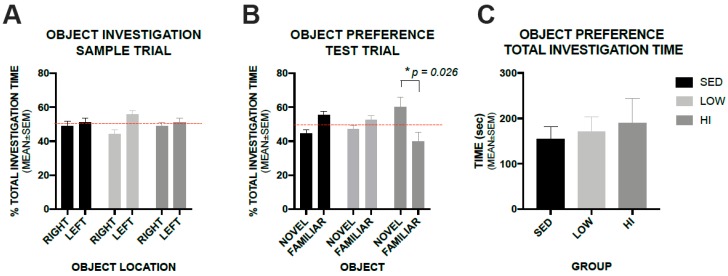
High-intensity exercise training increased preference for the novel object as shown by an increased percentage of investigation time compared to the familiar object, but low-intensity exercise training does not. Panel **A** shows percent of total investigation time for each group between similar objects placed on the right and left sides of the test area. Panel **B** shows percent of total investigation time between novel and familiar objects when one of the similar objects was replaced by a novel object. Dashed line represents 50% or equal preference. Panel **C** shows total investigation time during the test trial. Values represent the mean ± s.e.m.

A Novel Object Recognition test was utilized to assess recognition memory. During the Novel Object Recognition sample trial, the preference for similar objects placed on the right and left sides of the test area did not differ within groups (Figure 5A). When one of the objects was replaced with a novel object, the HI group mice spent a greater percentage of time investigating the novel object than the familiar object (Two-way Repeated-measures ANOVA shows significant interaction between GROUP and OBJECT (F(2,22) = 6.07, *p* = 0.0076) with post-hoc comparisons revealing that mice in the HI group spent significantly more time investigating the novel object compared to the familiar (*p* = 0.0283), while mice in the LOW and SED groups did not (*p* = 0.99 and *p* = 0.28, respectively) (Figure 5B). There was no difference in total investigation time among groups during the novel object test trial (One-way ANOVA F(2,22) = 0.197, *p* = 0.822) (Figure 5C).

## 4. Discussion

The build-up of amyloid plaque in the extracellular space of the brain resulting from the aggregation of the amyloid-β peptide is a pathological hallmark of AD. Soluble interstitial fluid Aβ concentration is a major contributing factor to its aggregation into amyloid plaque. This concept is supported by studies indicating that areas of the brain developing high plaque load late in life have high interstitial fluid Aβ levels early in life and, conversely, areas with low early life soluble Aβ levels have low late life plaque load [22,23].

We previously demonstrated that exercise training, in the form of treadmill running, administered early in life, from 3 to 6 months of age, lowered soluble Aβ_40_ and Aβ_42_ levels in the cortex and hippocampus of Tg2576 mice in an intensity dependent manner [25]. The reduction in soluble Aβ levels was associated with an intensity dependent upregulation of a number of Aβ clearance proteins. This study extends those findings and demonstrates that when the exercise training regimen is continued until late in life amyloid plaque levels in the cortex and hippocampus are also decreased in an intensity dependent manner. These two studies clearly demonstrate the benefits conferred by exercise training on amyloid pathology associated with AD are intensity dependent over the range investigated.

Ultimately the debilitating consequence of AD is the loss of cognitive function associated with neuronal dysfunction and loss, which is initiated by a cascade of events starting with the aggregation of soluble Aβ into oligomers and insoluble amyloid plaque [26]. Therefore, the hope is that reducing soluble Aβ levels early in life will result in reduced plaque later in life leading to increased cognitive function when compared to similar-aged sedentary individuals. Here, we demonstrate through behavioral tests of spatial and recognition memory that exercise training preserves cognitive function in Tg2576 mice above that of sedentary mice in an intensity dependent manner. The spatial memory effect was robust in the high-intensity exercise trained group as they significantly outperformed both the low-intensity and sedentary groups in all aspects of the Morris Water Maze test (time to learn platform location in acquisition trials, time to reach platform on final day of acquisition trial, initial latency to platform location in probe trial, and time in target quadrant in probe trial). The low-intensity group outperformed the sedentary group only in the initial latency to the platform location in the probe trial of the Morris Water Maze test, but not in any other aspects of MWM. The recognition memory performance differences assessed in NOR were not as robust as spatial memory. The HI group spent significantly more time investigating the novel object compared to the familiar object than the LOW and SED groups, but there was no difference between novel and familiar object investigation times in either the LOW or SED groups. These data indicate that chronic physical activity has the ability to prevent accumulation of Aβ. However, there may be a threshold for which lowered Aβ needs to reach to prevent memory decline. It is also possible that higher intensity exercise provides a broad range of positive effects on brain health that translate into improvement in memory function. Further studies are needed to determine the exact threshold of exercise that needs to be achieved to ensure robust memory preservation, as well as identify other factors involved in preventing memory decline in this model.

Chronic physical activity has long been known to confer health benefits and reduce risk of many chronic diseases including cardiovascular disease [27,28,29], type II diabetes [30,31], osteoporosis [32,33], depression [34,35], and breast and colon cancers [36,37]. Only within the past decade and a half has it been evaluated as an intervention against the onset and progression of AD in transgenic mouse models. Studies investigating chronic physical activity effects on AD using transgenic mouse models have varied widely in intensity, length of treatment, age at beginning and end of treatment, and whether or not the activity was voluntary of forced. While the majority of these studies have supported the concept that chronic physical activity is beneficial to improving AD outcomes, the results have been equivocal.

Considerable debate has focused on the voluntary or involuntary nature of the physical activity employed in previous studies, as forced running in mice has been criticized as being a stressful intervention [38], which may exacerbate AD symptomology. This contention is not without merit as psychological stress has been shown to increase Aβ levels in AD transgenic mice [39,40,41,42]. Wheel running has been utilized as an exercise mode to eliminate the stressful component of the activity as it allows the mouse to self-select running speed. The results of Yuede et al. [15] support the notion that stress does negate some of the positive effects of chronic physical activity on amyloid plaque load and cognitive function. When a wheel running group was intensity/duration/frequency matched to a treadmill running group the voluntary runners had significantly lower plaque counts and had better recognition memory than the forced runners. The forced runners did have a lower plaque count, but did not perform better on the recognition memory test than sedentary controls indicating that stress associated with forced running does not outpace the benefits associated with chronic physical activity. Yuede et al. [15] is currently the only study to directly compare voluntary and forced exercise as all other studies investigating the effects of chronic physical activity on AD related outcomes used only one exercise mode.

Results from wheel running studies vary as some demonstrated improved amyloid plaque load and cognitive function [15,43,44,45,46,47], others find no improvement in either plaque load or cognitive function [48,49,50], while still others find improvement in cognitive function, but no improvement in plaque load [51,52]. Results from treadmill running studies are more consistent with most finding improved amyloid plaque load and cognitive function [9,10,11,12,13,14,19,21], only Gimenez-Llort et al. [16] found no improvement in either plaque load or cognitive function, while Cho et al. [19] found an increase in cognitive function with no improvement in plaque load and Yuede et al. [15] found an improvement in plaque load, but not improvement in cognitive function. Collectively, evidence suggests that both wheel running and treadmill running have positive impacts on AD outcomes with treadmill running more consistently demonstrating this concept. In addition to exercise mode a major difference between wheel running and treadmill running studies is the intensity of the running. Mean distance reported in most wheel running studies would equate to average running speeds of approximately 2–6 m/min, whereas in treadmill running studies running speeds are typically between 10 and 15 m/min. Interestingly, the only treadmill running study to not find improvement in either amyloid plaque load or cognitive function was that of Gimenez-Llort et al. [16] which used a running speed of 4.2 m/min, much lower than all the other treadmill running studies which used running speeds of at least 10 m/min.

Previous studies investigating the effects of chronic physical activity regimens on amyloid plaque load and cognitive function imply that exercise intensity is an important factor in determining whether or not, and to what degree, improved AD outcomes occur. This study is the first to investigate the effects of long-term exercise training beginning early in life and continuing through midlife and provides clear evidence that high-intensity exercise training results in lower amyloid plaque load and greater cognitive function than low-intensity exercise training. This extends our previous study [25] and indicates that the decreased plaque load is the result of the intensity dependent decrease in Aβ_40_ and Aβ_42_ levels resulting from exercise training beginning early in life.

A question that arises is how do running speeds used for mice translate to humans? Obviously, it is difficult to arrive at this relationship with precision due to the many differences between mice and humans, including factors such as body size and the fact that mice are quadrupeds and humans are bipeds. In preliminary experiments, we discovered that the maximum running speed our Tg2576 mice could maintain on the treadmill at a 10% grade for two minutes was 53 m/min and they could tolerate 60% of that speed (32 m/min) for one hour well, but not much more. For that reason, we chose it for our HI group condition. We chose 15 m/min on a level treadmill for our LOW group condition because that corresponded to literature values for treadmill studies in transgenic AD mouse models close to the speed that appeared to be the threshold for improvement in many of the AD pathology parameters assessed [2]. To translate these running conditions to humans we consulted an individual who spent many years conducting an adult fitness program for healthy individuals from 20 to 70 years of age (Dr. Thomas Woodall, Eastern Illinois University) and posed the question, what is the maximum pace an average 30–40 year old male in your program could maintain for two minutes? He concluded it was about 5.5–6.0 min/mile. Sixty percent of that pace is approximately 9–10 min/mile which corresponds reasonably well to a fairly strenuous health related exercise training regimen in humans. The LOW group running speed of 15 m/min on a level treadmill is approximately 25% of the intensity of the two-minute maximum condition of our mice. This corresponds to a speed of 3.0 mph in humans which is a typical walking speed.

The question of whether or not there is an exercise training intensity ceiling or floor is yet to be determined. The fact that several studies employing low-intensity exercise regimens [16,49] do not report decreased plaque load or improved cognitive function suggests it is likely that an intensity threshold exists below which benefits of chronic physical activity do not occur. It is possible that the intensity-related rate of increase in exercise training benefit decreases and even reaches saturation as exercise intensity reaches very high levels. Further questions yet to be addressed include the impact of duration and frequency of exercise training regimens on AD outcomes and whether or not the effects of exercise training early in life persist even if the exercise training is discontinued.

## 5. Conclusions

In conclusion, 12 months of exercise training in the form of treadmill running, decreases amyloid plaque load in an intensity dependent manner in the Tg2576 mouse model of Alzheimer’s disease.High intensity exercise training robustly improves spatial and recognition memory to a greater degree than low intensity exercise training. Low intensity exercise training, at the level utilized in this study may provide some subtle improvements in spatial memory, but not in recognition memory relative to sedentary mice. These data support the concept that exercise training is an environmental intervention that reduces the risk of AD pathology in an intensity dependent manner.

## Abbreviations

Ab	Beta amyloid fragment of the amyloid precursor protein
AD	Alzheimer’s disease
CS	Citrate synthase
DAB	3,3’-diaminobenzidine
ET	Exercise training
HI	High-intensity exercise group
ISF	Interstitial fluid
LOW	Low-intensity exercise group
MWM	Morris Water Maze
NOR	Novel Object Recognition
SED	Sedentary group
